# Calibration and validation of modeled 5-year survival predictions among people with cystic fibrosis treated with the cystic fibrosis transmembrane conductance regulator modulator ivacaftor using United States registry data

**DOI:** 10.1371/journal.pone.0283479

**Published:** 2023-04-12

**Authors:** Lisa J. McGarry, Zahra Bhaiwala, Andrea Lopez, Conor Chandler, Christopher G. Pelligra, Jaime L. Rubin, Theodore G. Liou

**Affiliations:** 1 Vertex Pharmaceuticals Incorporated, Boston, MA, United States of America; 2 Evidera, Waltham, MA, United States of America; 3 Evidera, Bogotá, Colombia; 4 Adult CF Center, University of Utah, Salt Lake City, UT, United States of America; Cincinnati Children’s Hospital Medical Center, UNITED STATES

## Abstract

**Objectives:**

Cystic fibrosis (CF) is a rare genetic disease characterized by life-shortening lung function decline. Ivacaftor, a CF transmembrane conductance regulator modulator (CFTRm), was approved in 2012 for people with CF with specific gene mutations. We used real-world evidence of 5-year mortality impacts of ivacaftor in a US registry population to validate a CF disease-progression model that estimates the impact of ivacaftor on survival.

**Methods:**

The model projects the impact of ivacaftor vs. standard care in people with CF aged ≥6 years with *CFTR* gating mutations by combining parametric equations fitted to historical registry survival data, with mortality hazards adjusted for fixed and time-varying person-level characteristics. Disease progression with standard care was derived from published registry studies and the expected impact of ivacaftor on clinical characteristics was derived from clinical trials. Individual-level baseline characteristics of the registry ivacaftor-treated population were entered into the model; 5-year model-projected mortality with credible intervals (CrIs) was compared with registry mortality to evaluate the model’s validity.

**Results:**

Post-calibration 5-year mortality projections closely approximated registry mortality in populations treated with standard care (6.4% modeled [95% CrI: 5.3% to 7.6%] vs. 6.0% observed) and ivacaftor (3.4% modeled [95% CrI: 2.7% to 4.4%] vs. 3.1% observed). The model accurately predicted 5-year relative risk of mortality (0.53 modeled [0.47 to 0.60] vs. 0.51 observed) in people treated with ivacaftor vs. standard care.

**Conclusions:**

Modeled 5-year survival projections for people with CF initiating ivacaftor vs. standard care align closely with real-world registry data. Findings support the validity of modeling CF to predict long-term survival and estimate clinical and economic outcomes of CFTRm.

## Introduction

Cystic fibrosis (CF) is a genetic disease affecting more than 80,000 people worldwide [1–3]. CF is caused by reduced quantity and/or function of the CF transmembrane conductance regulator (CFTR) protein and affects multiple organ systems [1, 3, 4]. The most characteristic manifestation of CF is progressive loss of lung function, leading to early death [4, 5]. The survival of people with CF in the United States has gradually increased over the past 4 decades; median predicted survival for newborns with CF in the United States increased from approximately 29 years in 1988 to 48.4 years in 2019. This period saw the widespread adoption of newborn screening, improvements in standard care, and availability of CFTR modulator (CFTRm) treatments [5]. Standard care for CF in the United States includes physical airway clearance therapy, bronchodilators, inhaled mucolytics and antibiotics, a high-calorie, high-fat diet, and pancreatic enzyme replacement therapy in patients with pancreatic insufficiency [6–8].

Ivacaftor (brand name Kalydeco^®^, manufactured by Vertex Pharmaceuticals) is a CFTRm that facilitates chloride transport by potentiating the channel-open probability (or gating) of the CFTR protein at the cell surface [9, 10]. The US Food and Drug Administration approved ivacaftor in January 2012 for individuals aged ≥6 years with ≥1 copy of the *G551D-CFTR* mutation [11]; ivacaftor has subsequently been approved for those aged ≥4 months with ivacaftor-responsive genotypes [12]. Clinical studies have demonstrated that ivacaftor substantially improves lung function, nutritional parameters, and quality of life, and reduces frequency of pulmonary exacerbations [10, 13–16]. Moreover, real-world data have documented the benefits of treatment with ivacaftor, both over the short term as well as with prolonged use up to 5 years [17]. A recently published registry study evaluating real-world ivacaftor impacts in the United States and the United Kingdom over a 5-year time horizon shows reduced mortality and lung transplantation in people with CF treated with ivacaftor vs. those not treated with a CFTRm [18].

CF is a progressive disease and only lifetime follow-up can fully quantify the impact of CF treatments on survival. However, prior to the availability of such long-term data, simulation models can provide projections of the population-level effects of treatment to guide health policy and disease-management decisions [19–21]. Pharmacoeconomic simulation models are generally developed before product launch based on registration trial data with limited follow-up [22]. Model design and estimation require clinical judgment; the validity of model predictions cannot be tested until long-term real-world data become available to be used as a benchmark [23]. The CF patient-level simulation model (CF-PSM) has been developed for ivacaftor and used to project the impact of therapy on both survival and economic outcomes [24]. This model’s structure has been refined over time and has been used to evaluate additional CFTRm, including lumacaftor/ivacaftor, tezacaftor/ivacaftor and ivacaftor, and, most recently, elexacaftor/tezacaftor/ivacaftor and ivacaftor [24–32]. The CF-PSM combines inputs from national CF registries, randomized controlled trials, and other published and publicly available data sources to estimate the survival impact of CFTRm [25].

To date, the CF-PSM structure and outputs have been validated in accordance with guidelines of the ISPOR-SMDM Modeling Good Research Practices Task Force, which specifies 5 types of validity: (1) face validity, (2) internal validity, (3) cross-validity, (4) external validity, and (5) predictive validity [33]. In the model’s initial development, subsequent refinement, and uses prior to this study, validity types 1 through 4 were established through input from independent clinical and economic experts and health technology assessment bodies. Validity of the model’s outputs has been established for use across a range of products and geographic adaptations by comparison with independently developed CF models and by peer review of the modeling approach in publications [25, 34, 35].

Years of accumulated real-world experience with ivacaftor in the United States now enables testing for predictive validity of the CF-PSM. Specifically, in this study, model projections from the CF-PSM were compared with outcomes observed among people treated with ivacaftor and a matched comparison group not treated with a CFTRm in the US Cystic Fibrosis Foundation Patient Registry (CFFPR) [18].

### Cystic fibrosis patient-level simulation model (CF-PSM)

**Fig 1:**
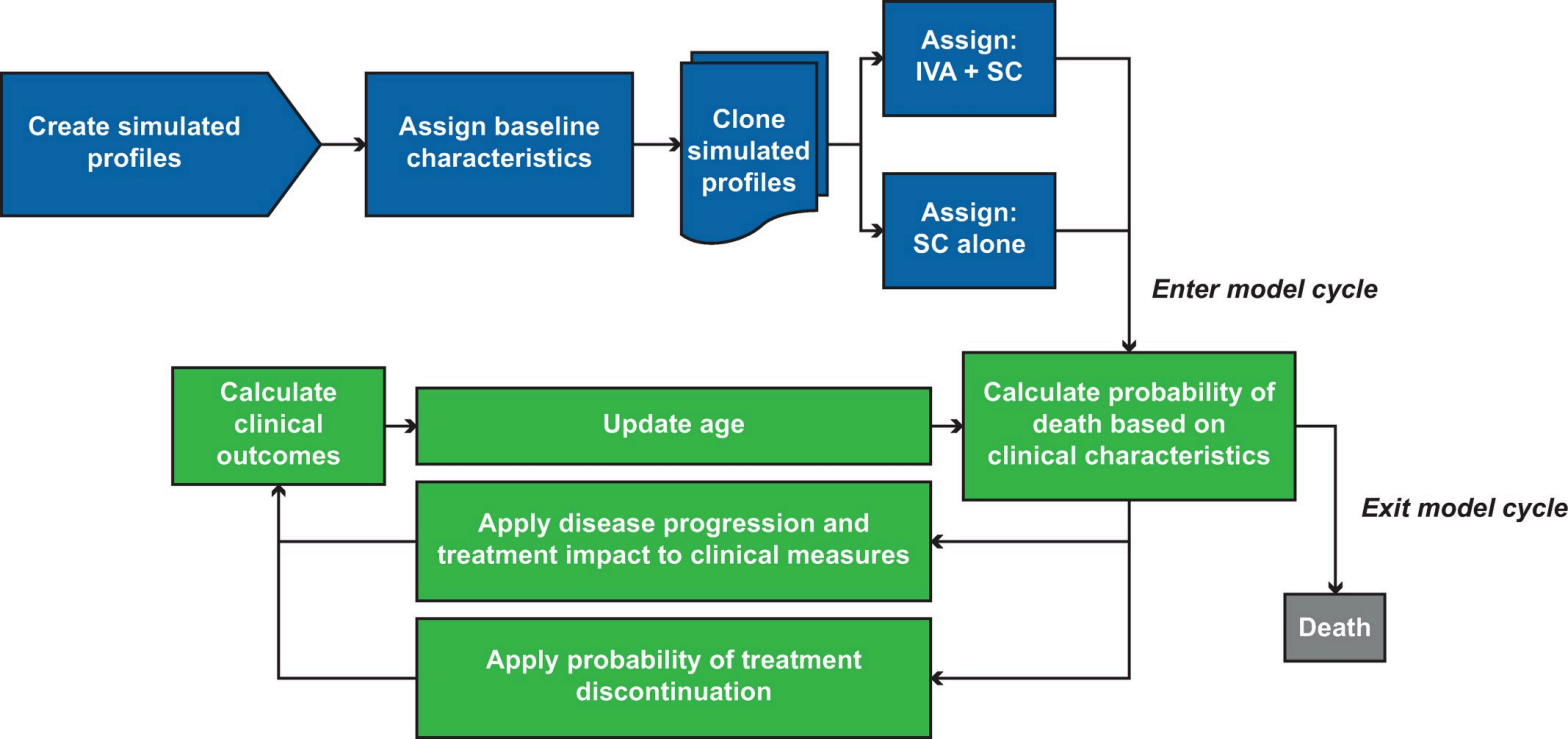
Model schematic for CF-PSM [25]. CF-PSM, cystic fibrosis patient-level simulation model; IVA, ivacaftor; SC, standard care. Adapted from Rubin JL, et al. Ther Adv Respir Dis. 2019;13:1753466618820186.

The CF-PSM was developed to project long-term clinical and economic outcomes of ivacaftor treatment based on data from randomized controlled trials and real-world sources that characterize CF survival, disease progression, and the efficacy of ivacaftor. While the model has been updated and adapted for subsequent CFTRm, the core model framework [25] (Fig 1) has remained consistent over time. The model’s structure, function, and estimation are described in detail in a recent publication of the predicted survival benefits of lumacaftor/ivacaftor in people with CF in the United States [25].

Briefly, the model estimates a simulated individual’s survival by combining age-specific estimates of the survival of individuals in the US CFFPR (1992 to 2011 birth cohorts) who received standard care but not a CFTRm, with a Cox proportional hazards (CPH) equation developed from US CFFPR data by Liou et al. in 2001 that links 5-year survival in CF to several key demographic and clinical characteristics [25, 36]. The lifetime survival in CFTRm-untreated people with CF was derived by fitting parametric equations to Kaplan–Meier data on overall survival from the observed CF population to generate a reference survival curve. The Liou CPH equation is applied to the reference survival curve at baseline to calculate the mortality hazard for each individual based on the 9 characteristics found to be predictive of survival: age, sex, pancreatic sufficiency, diabetes, respiratory infections (*Staphylococcus aureus*, *Burkholderia cepacia*), percent predicted forced expiratory volume in 1 second (ppFEV_1_), annual number of acute pulmonary exacerbations (PEx) requiring treatment, and weight-for-age z-score [25, 36]. The mortality hazard is recalculated in each model cycle by adjusting for changes in these characteristics over time, and each individual is simulated until death [25]. The impact of ivacaftor treatment on survival is modeled indirectly via its impact on lung function in terms of acute change in ppFEV_1_, reduction in the rate of ppFEV_1_ decline over time, and reduction in the number of PEx requiring treatment, as well as improvement in nutritional status in terms of weight-for-age z-score (Table 1) [10, 13, 14, 35, 37–40]. While the model appeared to provide plausible estimates of the lifetime impact of ivacaftor, additional investigation was warranted to confirm the predictive validity of combining the Liou CPH equation with the reference survival curves to model the survival impact of both disease progression and ivacaftor treatment.

**Table 1:** Model inputs for disease progression and ivacaftor treatment efficacy [10, 13, 14, 35, 37–40].

Parameter	Time period	Standard care alone	Ivacaftor initiated during ages 6 to 11 years	Ivacaftor initiated during ages ≥12 years
Acute change in ppFEV_1_ from baseline	Weeks 0 to 48	0^a^	+10.0 percentage point increase [14]	+10.5 percentage point increase [10]
Annual change in ppFEV_1_	Weeks 48+	Age-specific rates [38, 39]	47.1% reduction relative to standard care alone [40]	
		Ages 6 to 8: -1.12	Ages 6 to 8: -0.59	
		Ages 9 to 12: -2.39	Ages 9 to 12: -1.26	
		Ages 13 to 17: -2.34	Ages 13 to 17: -1.24	
		Ages 18 to 24: -1.92	Ages 18 to 24: -1.02	
		Ages ≥25: -1.45	Ages ≥25: -0.77	
Rate of PEx requiring IV antibiotics and/or hospitalization	Lifetime	Predicted rate is conditional on age and ppFEV_1_ [35, 37]	No impact assumed during ages 6 to 11; 55% lower rate relative to standard care alone during ages ≥12 years [10, 13]	55% lower rate relative to standard care alone [10, 13]
Acute change in weight-for-age z-score from baseline	Weeks 0 to 48	0^a^	+0.39 unit increase [14]	+0.33 unit increase [10]

IV, intravenous

PEx, pulmonary exacerbations

ppFEV_1_, percent predicted forced expiratory volume in 1 second.

^a^Ivacaftor clinical efficacy inputs are based on the placebo-controlled treatment effects observed in the randomized controlled trials. Because these effects are already placebo adjusted, people with cystic fibrosis treated with standard care alone are assumed to experience no change from baseline over the first 48 weeks of the model simulation.

### Ivacaftor post-authorization safety study

The ivacaftor post-authorization safety study had a follow-up time of 5 years, allowing for evaluation of the effects of ivacaftor treatment on disease progression and clinical outcomes [18]. The study was conducted using US CFFPR data from 2012 through 2016 and UK Cystic Fibrosis Registry data from 2013 through 2016 [18]. In the US disease-progression cohort, which was used in this model-validation exercise, Volkova et al. analyzed 805 people with CF aged ≥6 years who initiated ivacaftor treatment in 2012 as well as 3815 people with CF not eligible for ivacaftor (the only CFTRm available in 2012); the populations were matched on age, sex, and disease severity (by *CFTR* genotype) [18]. Longitudinal evaluation found that, relative to the comparator cohort, ivacaftor-treated individuals had significantly better-preserved lung function (as assessed by ppFEV_1_), improved nutritional status, lower frequencies of PEx and hospitalizations, lower probability of lung transplant, and higher probability of transplant-free survival over up to 5 years of follow-up [18].

### Study objectives

The objective of the current analysis was to assess the predictive validity of the CF-PSM by comparing outputs of this simulation model over a 5-year horizon with real-world 5-year survival data from the US CFFPR as reported in the Volkova et al. study [18].

## Methods

Our examination of predictive validity focused first on the prediction of survival in the CF population not receiving CFTRm and then on the impact of ivacaftor on survival; we intended to examine and revise methods as necessary to replicate observed data. Several model inputs and assumptions were of particular interest for evaluation due to their importance in estimating survival, including accounting for individuals’ characteristics at baseline, the estimation of the reference survival curve for CFTRm-untreated people with CF, the use of the CPH equation [41] to adjust for individual characteristics and estimate survival in CFTRm-treated and -untreated people with CF, and the estimation of the real-world survival effect of ivacaftor from clinical efficacy among randomized controlled trial participants.

### Validation process

The validation process comprised a stepwise approach. First, de-identified baseline profiles for the 805 individuals treated with ivacaftor in the 5-year disease-progression cohort from Volkova et al. [18] were run through the model. Each patient profile comprised the 9 characteristics that Liou et al. had found to be predictive of survival in CF. The 5-year model projections for the cohort receiving standard care alone were compared with the observed 5-year survival in the Volkova study for people with CF who were ineligible for ivacaftor to ensure that the combination of the background survival curve and the CPH equation was appropriately capturing the impact of disease progression on survival in the absence of CFTRm. Finally, the 5-year survival estimate for the modeled ivacaftor-treated cohort was generated and compared with the observed 5-year survival in ivacaftor-treated individuals from the Volkova study to evaluate the prediction of ivacaftor impact using the CPH equation and ivacaftor clinical efficacy data. Probabilistic sensitivity analyses via Bayesian Monte Carlo simulation [25] generated the 95% credible intervals (CrIs) surrounding the point estimates of the survival predictions from the best-fit scenario for ivacaftor mortality risk, standard care mortality risk, and mortality relative risk from the CF-PSM.

### Ethics

This study was reviewed and exempted by the Advarra Institutional Review Board (IRB). The IRB waived the requirement for informed consent.

## Results

Among the 805 baseline individual patient profiles from the Volkova study provided by the US CFFPR, 178 observations (22.7%) were missing 1 or more covariate values. Where sufficient data were available, missing baseline values were imputed using multiple imputation by chained equations, in which values for missing covariates are imputed using non-missing covariates [42, 43]. This imputed data set of 779 individuals was used to conduct base-case analyses for the model validation study (Table 2) [18].

**Table 2:** Demographics and baseline characteristics for validation cohort from the Volkova study^a^ [18].

Baseline characteristic	Modeled validation cohort^a^ (*n* = 779)
Age, mean (SD), years	19.6 (11.8)
Male, %	50.1
Weight-for-age z-score, mean (SD)	0.1 (1.3)
ppFEV_1_, mean (SD), percentage points	76.6 (24.7)
Annual PEx rate, mean (SD)	0.7 (1.0)
Pancreatic sufficiency, %	5.20
Diabetes mellitus, %	17.3
*Burkholderia cepacia* positive, %	2.20
*Staphylococcus aureus* positive, %	66.0

PEx, pulmonary exacerbations

ppFEV_1_, percent predicted forced expiratory volume in 1 second

SD, standard deviation.

^a^Subset of patient profiles from the US disease-progression cohort of the ivacaftor post-authorization safety study [18] with complete baseline data after imputation.

When comparing the 5-year survival in the modeled CFTRm-untreated cohort with the observed 5-year survival in ivacaftor-ineligible individuals from the Volkova study, we initially found that the CF-PSM over-predicted mortality in the CFTRm-untreated group by over 5 years (17% modeled vs. 6.0% observed). On close examination, we found that calculating the baseline mortality risk for each simulated patient based on individual clinical characteristics at baseline using the CPH equation and reference survival curve identified individuals with extremely high mortality hazards at baseline due to concurrent risk factors and the multiplicative nature of CPH. A revision was consequently made to instead assign all people with CF an age-specific mortality risk at baseline derived from the reference survival curve and not to adjust for baseline clinical characteristics. Instead, the CPH equation was used only to adjust mortality hazard in each subsequent post-baseline model cycle. This revision improved model fit, but the model continued to slightly over-predict 5-year mortality in CFTRm-untreated people with CF (9.7% modeled vs. 6.0% observed). To more closely match the observed 5-year mortality rate in CFTRm-untreated individuals from the US CFFPR, the reference survival curve was shifted to increase the median survival from 39.7 years, which reflected survival in the historic population from which the survival curve was derived [25], in full-year increments to 45 years. Following this calibration, the modeled 5-year mortality projections closely approximated real-world data for CFTRm-untreated people with CF (6.4% modeled [95% CrI: 5.3% to 7.6%] vs. 6.0% observed).

**Fig 2:**
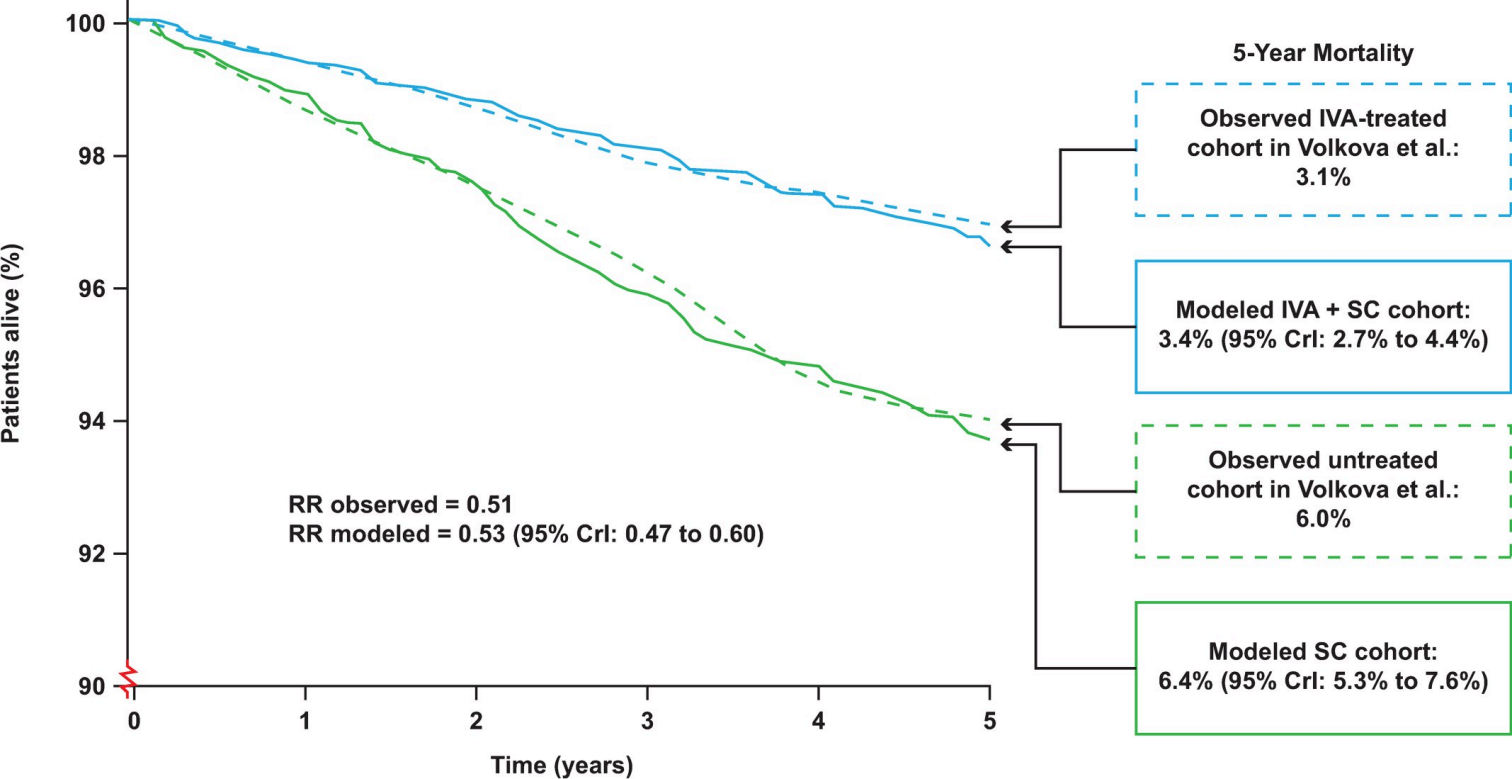
Comparison of observed and modeled 5-year mortality benefits of ivacaftor. CrI, credible interval; IVA, ivacaftor; RR, relative risk; SC, standard care.

We next generated model survival projections for an ivacaftor-treated cohort and found that the CF-PSM accurately replicated the observed ivacaftor treatment effect in the post-authorization safety study, estimating mortality of 3.4% (95% CrI: 2.7% to 4.4%) vs. the observed 3.1% [18]. The model also predicted that those treated with ivacaftor had a 5-year relative risk of mortality vs. those who were CFTRm-untreated of 0.53 (95% CrI: 0.47 to 0.60), similar to the 0.51 observed in the Volkova study [18] (Fig 2).

## Discussion

CFTRm such as ivacaftor are projected to change the trajectory of CF disease and extend survival by upwards of 30 years based on a published patient-level lifetime simulation model [24–32]. With the availability of real-world survival data from a recent post-authorization safety study that evaluated the long-term treatment benefits of ivacaftor using data from the US CFFPR over 5 years (2012 to 2016) [18], we were able to test the predictive validity of the simulation model. We found that, after calibration, modeled projections closely matched real-world survival data for both ivacaftor-treated and CFTRm-untreated people with CF over 5 years.

To validate the CF-PSM, we used a stepwise approach in which we first ensured that the model accurately predicted survival in the absence of CFTRm treatment. Consistent with modeling guidelines, we calibrated the model to achieve the best fit by adjusting inputs to replicate real-world target data as closely as possible [44]. Removing the CPH adjustment from the calculation of the baseline mortality hazard and applying it only to subsequent post-baseline model cycles, combined with adjusting the estimate of median survival in the reference population, led to an excellent model fit. With these adjustments, modeled 5-year mortality projections closely approximated real-world outcomes in the untreated and ivacaftor-treated CF populations. After calibration, the CF-PSM predicted that, over 5 years, people treated with ivacaftor had a relative risk of mortality of 0.53 vs. CFTRm-untreated people with CF, similar to the 0.51 relative risk observed in the 5-year Volkova study [18].

A key finding of this exercise is that, although the Liou CPH equation was developed in a population entirely unexposed to CFTRm, the changes in clinical outcomes as a result of CFTRm treatment combined with the CPH equation generate 5-year survival estimates that closely match the impact of ivacaftor observed in the real world. This result suggests that models using this equation and applying similar methods can provide plausible estimates of treatment impact and outcomes, and can be used with confidence to extrapolate beyond clinical trial data to provide informative predictions of long-term benefits of CF treatments, based on currently available evidence.

The change in the baseline application of the Liou CPH equation was an unexpected outcome of the calibration process. Prior to this validation study, the CF-PSM adjusted an individual’s baseline age-specific mortality hazard with reference to the average characteristics of people with CF in the background population (ie, the 2011 US CFFPR population from which the reference values used in the Liou CPH equation were derived) [45]. This baseline mortality hazard adjustment was intended to account for differences in individual characteristics at baseline vs. the Liou reference population in order to improve model predictions when the modeled individual was “healthier” or “sicker” than the average person with CF at baseline. However, because age-specific baseline characteristic summary measures were unavailable for the reference population (eg, mean ppFEV_1_ for every age), all modeled baseline hazards were calculated relative to the reference population averages (eg, mean ppFEV_1_ for the entire population, across all ages). This approach led to many younger-than-average modeled people with CF appearing unusually healthy while those older than average appeared unusually ill, with their age-specific hazards adjusted accordingly. Moreover, when individual baseline hazard predictions were examined, it was apparent that, for a small number of people with CF who had multiple risk factors at baseline, the CPH model generated extremely high mortality hazards at baseline due to the multiplicative nature of the CPH model. Removing this adjustment assumes that the baseline hazard for modeled people with CF is the same as that for people with CF in the reference population of the same age. This simplification resulted in model output that was a much better fit to the observed 5-year US CFFPR mortality rates.

We further note that the increase in median survival for the reference survival curve as a result of calibration is consistent with epidemiologic data from the US CFFPR that show an increase in median survival over the past several decades. The predicted life expectancy from the most recent registry report [5] is based on a small number of yearly deaths and is derived from a data set that includes a substantial number of people with CF on CFTRm; therefore, it is not an estimate for a wholly CFTRm-untreated population and cannot be used in the CF-PSM. However, by calibrating our estimate of median survival to the Volkova data for CFTRm-untreated people with CF only, we were able to provide additional insight into survival gains in the hypothetical CFTRm-untreated population while also ensuring that the calibration was not biased to show desired treatment outcomes, since no calibration was performed for the treated people with CF. In addition, our observation that it was necessary to increase the median survival to achieve model fit is consistent with the recent re-analysis of CFFPR data by Liou and colleagues [46]. By replicating the analyses using more recent registry data, Liou et al. showed that, while the relationship between risk factors and survival among those in the US CFFPR has not changed over time, the average survival (intercept) in CFTRm-untreated people with CF had to be increased to fit more recent mortality patterns [46].

Because calibration changed the 5-year survival estimate in modeled people with CF treated with ivacaftor and standard care, we re-examined previous published analyses in which the CF-PSM was used to assess outcomes among populations with a range of ages over a lifetime horizon [25]. When analyses were replicated using the calibrated model, the prediction error introduced by the baseline hazard adjustment had minimal impact on lifetime projections of treatment benefit because the individuals with very high mortality hazards died in early model cycles. Moreover, possible errors due to imprecise estimation of baseline hazard (inflated hazards in older individuals, deflated hazards in younger individuals with CF) generally averaged out across the CF population. So, while the imprecision was evident over shorter time horizons (ie, 5 years), it had minimal impact over longer time horizons (ie, lifetime). In addition, underestimation of median survival based on the pre-calibration reference curve similarly affected both individuals treated with ivacaftor and those receiving standard care. Estimated survival in both treatment cohorts improved due to the increase in the assumed median age at death, with a modest increase in incremental survival for ivacaftor-treated individuals, suggesting that previous versions of the model may have slightly underestimated the survival benefit of ivacaftor treatment. We, therefore, concluded that although removal of the baseline hazard adjustment was necessary for the model to provide valid survival estimates over the shorter time horizon of 5 years, the calibration did not meaningfully change lifetime estimates of treatment benefit or conclusions regarding the impact of ivacaftor treatment that have been previously reported.

While the availability of published data from a large real-world data set provided the unique opportunity to validate the CF-PSM, our study is subject to a number of limitations. The validation sample comprising individuals with complete baseline profiles used in the model was a subset of the ivacaftor-treated cohort in the Volkova study (779 of 805 available profiles; 97%); although baseline characteristics were similar to the overall sample, any bias introduced by using this subset is unknown. Similarly, bias introduced by imputation of missing data cannot be quantified. In addition, although the comparator group in the Volkova study was matched to treated individuals at baseline, loss of individuals from the comparator arm due to availability of a novel CFTRm may have selected for remaining individuals who were healthier at baseline. Thus, underestimation of the ivacaftor survival benefit used as a validation target may have occurred. Considering the close match between the treatment effect in Volkova et al. and that of the calibrated model, this would suggest that any underestimation of the ivacaftor survival benefit in Volkova et al. may also be present in the model.

We further note that the available data for validation included only 5 years of follow-up in a US population of people with CF treated with a single CFTRm; however, Liou et al. demonstrated that the model developed in a historical population accurately predicted survival over a 25-year study period, despite changes in standard care and expected survival over this period. It will be of interest to continue validating and refining the model as longer-term data and real-world data for additional CFTRm and broader populations become available.

Finally, the CF-PSM structure is flexible to incorporate additional evidence as it becomes available for new treatment modalities, long-term adherence, and durability of clinical benefits, which are likely to impact the clinical characteristics, such as ppFEV_1_, that drive CF-PSM projections. However, because the median survival in CF is now predicted to be >50 years and therapeutic options and standard care are rapidly evolving in CF, the scope of the current CF-PSM may be subject to future revision. Changes may be needed to incorporate as yet unknown lifetime impacts, such as differential outcomes for subgroups, positive or negative effects of long-term treatment on non-respiratory organ systems, and adverse outcomes of CF in an aging population. These remain rich areas for future study.

This study demonstrates the successful validation of a long-term survival model in CF by comparing modeled results to real-world, observed, 5-year survival in a US registry. These results show that, with appropriate model assumptions, disease progression and CFTRm clinical efficacy data in combination with the Liou CPH equation provide valid estimates of long-term survival and impact of CFTRm. The validation of the modeling methods, including use of the Liou CPH equation to estimate survival in both treated and CFTRm-untreated people with CF, should encourage confidence in lifetime projections of CF population survival, both for ivacaftor and for other CF treatments generated using similar modeling methods.

## Conclusion

After calibration, modeled 5-year survival projections for people with CF with gating mutations initiating ivacaftor and standard care tracked closely to trends observed in real-world registry data. These findings support the validity of modeling CF using the described approach to predict long-term survival and estimate clinical and economic outcomes attributable to CFTRm.
